# Efficacy of dexamethasone on postoperative analgesia in children undergoing hypospadias repair

**DOI:** 10.12669/pjms.321.9089

**Published:** 2016

**Authors:** Mehdi Shirazi, Hilda Mahmoudi, Behnam Nasihatkon, Sina Ghaffaripour, Ali Eslahi

**Affiliations:** 1 Dr. Mehdi Shirazi, Associate Professor of Urology, Department of Urology, Shiraz University of Medical Sciences, Shiraz, Iran; 2 Dr. Hilda Mahmoudi, Department of Anesthesiology, Anesthesiology and Critical Care Research Center, Shiraz University of Medical Sciences, Shiraz, Iran; 3 Dr. Behnam Nasihatkon, Urologist, Department of Urology, Shiraz University of Medical Sciences, Shiraz, Iran; 4 Dr. Sina Ghaffaripour, Associate Professor of Anesthesiology, Department of Anesthesiology, Anesthesiology and Critical Care Research Center, Shiraz University of Medical Sciences, Shiraz, Iran; 5 Dr. Ali Eslahi, Assistant Professor of Urology, Department of Urology, Shiraz University of Medical Sciences, Shiraz, Iran

**Keywords:** Dexamethasone, Post operative pain, Hypospadias

## Abstract

**Background and Objective:**

Management of post operative pain in children undergoing hypospadiasis repair, accounts for optimized surgery outcomes and improved patients’ satisfaction. Thus, various studies have widely investigated the best approaches for the pain management. In this study our aim was to determine the effect of dexamethasone in combination with penile nerve block on the postoperative pain and complications in the children undergoing hypospadias surgery.

**Methods:**

In this randomized double-blind placebo controlled trial, after obtaining informed consent from parents or legal guardians, 42 children undergoing surgical treatment of hypospadias were randomized in two groups to receive either IV dexamethasone 0.5 mg/kg (n=23) or placebo (normal saline) (n=19) during the operation. Penile block was performed in both groups using Bupivacaine 0.5% (1mg/kg) at the end of the procedure. By the end of the operation, FLACC (Face, Leg, Activity, Cry, Consolability) pain score was assessed as the primary outcome of the study. Secondary outcomes includes timing and episodes of rescue medication consumption, post operative nausea \vomiting and bleeding. All the outcomes were assessed in the recovery room and after 2, 6, 12, and 24 hours.

**Results:**

The median of FLACC pain scores at the recovery room and 2, 6, 12, and 24 hours post operation was 2, 1, 1, 1, and 2 for the dexamethasone group and 8, 8, 7, 7, and 8 for the placebo group respectively. This were significantly different (P<0.000). The median time of first rescue medication consumption was 8 hours post operation for the dexamethasone group and three hours for the placebo group which was significantly different (z= 4.57, p<0.000). The maximum episode of post operative rescue medication consumption in dexamethasone group was 4 episodes in only one patient and the minimum was one episode in 11 patients. In comparison numbers in placebo group were five episodes in seven patients and three episodes in four patients. The result indicated that there was statistically significant difference between two groups in terms of episodes of rescue medication consumption (Chi2= 31.4, p<0.000).

**Conclusion:**

Single dose of intravenous dexamethasone (0.5 mg/kg) in combination with penile block decreased the post operative pain measures, and total post operative analgesic requirement. It also increased the onset of the first analgesic requirement compared to penile block alone.

## INTRODUCTION

Hypospadias is an incomplete fusion of urethral folds in early fetal development. It is one of the most common anomalies of genitourinary tract in males^1^,^2^ and occurs in 17-33 per 10,000 newborns.^3^,^4^

Hypospadias repair surgery is an invasive procedure whose postoperative phase is very painful.^5^ Pain management can be counted as an important step in all surgeries, especially in pediatric and neonatal surgical procedures. It has been reported that up to 40% of the children undergoing surgeries suffer from moderate to severe post-operative pain.^6^ In addition to the significance of post-operative pain management in the quality of life and satisfaction of the patients, it plays a great role in minimizing the complications and improving the surgery outcome in certain types of surgeries. In hypospadias surgery, post-operative pain results in manipulation of wounds and consequent infection, hemorrhage, and wound dehiscence.^7^ These complications cause significant preventable morbidities. Post-operative pain management also alleviates the local and systemic inflammation resulting in more favorable outcomes.^8^

In general, various techniques, such as regional anesthesia and systemic medications, are used to provide postoperative analgesia. Nowadays, multimodal anesthetic techniques are preferred for postoperative pain management in infants and children.^2^,^9^

Dexamethasone is a powerful anti-inflammatory drug and its efficacy in reduction of postoperative pain in various surgical procedures^10^-^12^ and nausea as well as vomiting has been confirmed in some procedures, such as tonsillectomy and orchiopexy.^13^,^14^ It suppresses the tissue bradykinin and reduces the production of neuropeptide at the nerve endings resulting in pain relief.^15^

As far as we know, this is the first study to determine the effect of dexamethasone in combination with penile nerve block on the postoperative pain and complications in the children undergoing hypospadias surgery.

## METHODS

In a randomized double blind placebo controlled study, after obtaining informed consent from parents or legal guardians, we enrolled 42 children, aged between 1-6 years old who were candidate for hypospadiasis surgery. Patients were randomly assigned in to two groups by blocked randomization method. All the surgeries were performed by the first author. The study was approved by a local ethics committee.

After induction of general anesthesia, the patients of the experimental group (N=23) received 0.5 mg/kg dexamethasone, while the control patients (N=19) got the same volume of normal saline by an anesthesiologist who was blind to intervention assignment. Dexamethasone and normal saline were administered using similar encoded vials. After administration, the codes were recorded in data forms and decoded by a third colleague for statistical analysis.

All the patients underwent Tubularized Incised Plate (TIP) procedure as their repair method. At the end of the procedure, a penile block with Bupivacaine 0.5% (1mg/kg) was performed in both groups by the same anesthesiologist.

By the end of the operation, FLACC (Face, Leg, Activity, Cry, Consolability) pain score was assessed as the primary outcome of the study. If the pain score was greater than 8, intravenous fentanyl (2 microgram/kg) was administered. And if the pain score was between 4 and 8, acetaminophen 40 mg/kg rectally was administered as rescue medication. Secondary outcomes includes timing and episodes of rescue medication consumption, post operative nausea \vomiting and bleeding.

All the outcomes were assessed in the recovery room and after 2, 6, 12, and 24 hours by a blinded urology resident to treatment assignment. The FLACC scoring system is shown in Table-I.^16^ The data were collected and statistically analyzed in order to compare the outcomes in the case and the control group.

**Table-I:** FLACC scoring system Chart.

	Score
*Face*	
0-No particular expression or smile	
1-Occasional grimace or frown, withdrawn, disinterested	
2-Frequent to constant quivering chin, clenched jaw	
*Legs*	
0-Normal position or relaxed	
1-Uneasy, restless, tense	
2-Kicking, or legs drawn up	
*Activity*	
0-Lying quietly, normal position, moves easily	
1-Squirming, shifting back and forth, tense	
2- Arched, rigid or jerking	
*Cry*	
0-No cry (awake or asleep)	
1-Moans or whimpers; occasional complaint	
2- Crying steadily, screams or subs, frequent complaints	
*Consolability*	
1. Content, relaxed	
2. Reassured by occasional touching, hugging or being talked to, distractible
3. Difficult to console or comfort	

Total Score

### Statistical methods

Since, normal distribution assumption is not satisfied, Wilcoxon rank-sum test was used in order to compare the patients’ age, weight, time of first rescue medication consumption, and FLACC scores. Nausea/vomiting and episodes of rescue medication consumption were compared using Chi-square test. The results are presented as median for age, weight, time of rescue medication consumption, and FLACC scores. We used percent for nausea/vomiting and bleeding. Analyses were performed using STATA 13 for Windows. Bonferroni adjusted significance level of two-sided p value <0.006 (0.05/8) was calculated to account for the increased possibility of type one error.

## RESULTS

We recruited 23 patients in the dexamethasone group and 19 patients in the placebo group. The study results revealed no significant difference between the two groups regarding age and weight. The median time of first rescue medication consumption was 8 hours post operation for the dexamethasone group and 3 hours for the placebo group which was significantly different (z= 4.57, p<0.000).

The maximum episode of post operative rescue medication consumption in dexamethasone group was 4 episodes in only one patient and the minimum was one episode in 11 patients. The comparative numbers in placebo group were five episodes in seven patients and three episodes in four patients. The result indicated that there was statistically significant difference between two groups in terms of episodes of rescue medication consumption (Chi2= 31.4, p<0.000). Table-II depicts more details.

**Table-II:** Episodes of post operative rescue medication consumption in Dexamethasone and placebo groups.

	Episodes of post operative rescue medication consumption				

	1	2	3	4	5
Dexamethasone group	11	8	3	1	0
Placebo group	0	0	4	8	7

Chi2= 31.4, p<0.000.

The median of FLACC pain scores at the recovery room and 2, 6, 12, 24 hours post operation was 2, 1, 1, 1, 2 respectively for the dexamethasone group and 8, 8, 7, 7, 8 for the placebo group which were significantly different (P<0.000).

**Table-III:** Characteristics of the study population.

	Dexamethasone group	Placebo group	Z value	P-value*
Age (months), median	42	36	1.64	0.100
Weight (kg), median	15	14	0.96	0.360
FLACC r	2	8	-5.27	0.000
FLACC2	1	8	-5.43	0.000
FLACC6	1	7	-5.51	0.000
FLACC12	1	7	-5.53	0.000
FLACC24	2	8	-5.59	0.000
Nausea\Vomiting (%)	47.4	17.4	4.37**	0.03

* P values less than 0.006 (Bonferroni adjusted) consider statistically significant.

** Chi2 value.

Four patients in the dexamethasone group (17.4%) and nine patients in the placebo group (47.4%) experienced nausea/vomiting which was not statistically significant. (Chi2= 4.37, P=0.03). None of the study patients developed post operative bleeding.

## DISCUSSION

Dexamethasone has a powerful anti-inflammatory activity and its efficacy in reducing the pain and complications after oral surgery, spinal surgery, and orchiopexy has been proved by previous studies.

Post-operative pain is an important issue that may be undertreated in the pediatric population. The present study is one of the first studies on the effect of dexamethasone combined with penile block on post operative pain management in pediatrics. We demonstrated that a single dose of intravenous dexamethasone (0.5 mg/kg) in combination with penile block decreased the post operative pain, and total post operative analgesic requirement, and increased the onset of the first analgesic requirement compared to penile block alone.

Dexamethasone in combination with caudal block has been demonstrated to be effective in post-operative management of orchiopexy.^14^ It was also more effective in pain management compared to caudal block alone.

Dexamethasone has also been used for post operative analgesia after pediatric tonsillectomy. Hermans and his colleagues reached desirable results with a single dose of dexamethasone in this group. In addition, a decrease was observed in the incidence of nausea and vomiting and the patients’ pain score.^17^

Theoretically, steroids can decrease the pain perception since they are among the strong anti-inflammatory drugs.^18^ They have been shown to decrease pain after oral surgeries such as tonsillectomy,^10^ spinal surgery,^11^ and laparoscopic surgery.^12^ However, the exact mechanism of its analgesic effect has not been well understood yet. This effect might result from its suppression of tissue bradykinin or nerve ending neuropeptide production.^15^ On the other hand, steroids are able to inhibit cyclooxygenase isoform-2 production in the peripheral tissues and the central nervous system.^19^

Prolonged duration of action is another promising characteristic of dexamethasone. Hval and colleagues^20^ have demonstrated that the analgesic effect of dexamethasone was prolonged significantly for three days after administration when combined with a long-acting Non-Steroidal Anti-Inflammatory Drug (NSAID); i.e., rofecoxib. Even after dexamethasone clearance, its clinical effect will continue because it changes the DNA transcription to proteins. This explains why dexamethasone’s anti-inflammatory effects will continue even after its half life (6 hours) has passed.

In this study, we followed our patient for 24 hours after the surgery. During this time, a significant reduction was observed in the dexamethasone group’s pain scores (p-value<0.000) compared to the control group (only penile block with Bupivacaine).

Regarding the demographic issues of our study groups it is notable that the average age of patients undergoing hypospadias surgery in our country is higher than that is in the literature. The reason is partly explained by the cultural reasons in our country that most of parents are afraid of doing operations on their very young children.^21^

The side effects of opioids are more frequent in pediatric patients. Therefore, many centers have changed their method of analgesia to non narcotic medications in pediatric patients. Acetaminophen and NSAIDs are among these drugs. Up to now, dexamethasone has not been used frequently for post-operative pain management in pediatric urologic surgery. Moreover, the previous studies conducted on pediatric tonsillectomy patients have come to conflicting results. Some of them recommended dexamethasone^22^,^23^ and some did not. 24, 25 One of the reasons for these discrepancies is the drug dose and the total dosages used in different studies. In this study, we used dexamethasone 0.5 mg per kilogram of our patients’ weight without any maximum dose consideration. The discrepancies may also be due to the surgical and anesthetic techniques, additive analgesics, and lack of standardization for pain score and its management. We tried to standardize the pain score with FLACC score because most of our patients were less than three years old.

The side effects of preoperative single dose of dexamethasone have been reviewed in a meta-analysis by Waldron et al.^26^ in which, no statistically significant increase was found in the rate of post-operative infection. In addition, hyperglycemia has been reported in the first 24 hours after administration of a single dose of dexamethasone, which has no clinical implication.

In our study, 17.4% of patients in dexamethasone group and 47.4% of patients in placebo group experienced nausea/vomiting. Although the incidence are clinically different but we could not show a statistically significant difference because we used the conservative Bonferroni adjusted significance level of p value <0.006. The incidence is statistically higher in placebo group at p level of 0.05.

### Limitation of the study

The limitation of our study is that we only included patients with distal hypospadiasis which may decrease the generalizability of the results.

## CONCLUSION

Intra-operative administration of 0.5 mg/kg dexamethasone can effectively decrease the post-operative pain in the children undergoing hypospadias repair surgery.
